# FDG-PET/CT for pre-operative staging and prognostic stratification of patients with high-grade prostate cancer at biopsy

**DOI:** 10.1186/s40644-015-0038-0

**Published:** 2015-03-03

**Authors:** Jean-Mathieu Beauregard, Annie-Claude Blouin, Vincent Fradet, André Caron, Yves Fradet, Claude Lemay, Louis Lacombe, Thierry Dujardin, Rabi Tiguert, Goran Rimac, Frédérick Bouchard, Frédéric Pouliot

**Affiliations:** Division of Nuclear Medicine, Department of Radiology and Cancer Research Center, Université Laval, Quebec City, Canada; Division of Nuclear Medicine, Department of Medical Imaging and Oncology Axis of CHU de Québec Research Center, CHU de Québec, Quebec City, Canada; Division of Urology, Department of Surgery and Cancer Research Center, Université Laval, Quebec City, Canada; Division of Urology, Department of Surgery and Oncology Axis of CHU de Québec Research Center, CHU de Québec, Quebec City, Canada; Centre hospitalier du Christ-Roi, Quebec City, Canada

**Keywords:** FDG-PET/CT, High-grade, Prognostic stratification, Prostate cancer, Staging

## Abstract

**Background:**

The role of ^18^F-fluorodeoxyglucose positron emission tomography/computed tomography (FDG-PET/CT) in prostate cancer (PCa) has not been well defined yet. Because high-grade PCa tends to exhibit increased glycolytic rate, FDG-PET/CT could be useful in this setting. The aim of this study was to assess the value of FDG-PET/CT for pre-operative staging and prognostic stratification of patients with high-grade PCa at biopsy.

**Methods:**

Fifty-four patients with a Gleason sum ≥8 PCa at biopsy underwent FDG-PET/CT as part of the staging workup. Thirty-nine patients underwent radical prostatectomy (RP) and pelvic lymph node (LN) dissection, 2 underwent LN dissection only, and 13 underwent non-surgical treatments. FDG-PET/CT findings from clinical reports, blinded reading and quantitative analysis were correlated with clinico-pathological characteristics at RP.

**Results:**

Suspicious foci of increased FDG uptake were found in the prostate, LNs and bones in 44, 13 and 6% of patients, respectively. Higher clinical stage, post-RP Gleason sum and pattern, and percentage of cancer involvement within the prostate were significantly associated with the presence of intraprostatic FDG uptake (IPFU) (*P* < 0.05 in all cases). Patients without IPFU who underwent RP were downgraded to Gleason ≤7 in 84.6% of cases, as compared to 30.8% when IPFU was reported (*P* = 0.003). Qualitative and quantitative IPFU were significantly positively correlated with post-RP Gleason pattern and sum, and pathological T stage. Absence and presence of IPFU were associated with a median 5-year cancer-free survival probability of 70.2 and 26.9% (*P* = 0.0097), respectively, using the CAPRA-S prognostic tool.

**Conclusion:**

These results suggest that, among patients with a high-grade PCa at biopsy, FDG-PET/CT could improve pre-treatment prognostic stratification by predicting primary PCa pathological grade and survival probability following RP.

## Background

Staging and prognostication of primary prostate cancer (PCa) is of prime importance, especially for aggressive PCa, for which failure rate to local therapy is high [1]. Over the last decades, a number of clinical tools such as nomograms and imaging technologies have gained wide acceptance, but their accuracy for pre-treatment staging and prognostication is limited.

Molecular imaging with positron emission tomography/computed tomography (PET/CT) can detect molecular changes within cancer cells before morphological changes become apparent on conventional anatomical imaging such as standalone CT or magnetic resonance imaging [2]. The most widely used PET/CT application is the assessment of glucose metabolism using the glucose analogue ^18^F-fluorodeoxyglucose (FDG) [3]. FDG uptake has been shown to correlate with tumour grade and aggressiveness for many cancers, including metastatic PCa [4,5]. Nevertheless, FDG-PET/CT is not routinely performed in PCa patients, as low FDG accumulation in the majority of PCa tumours, which tend to be indolent, has been perceived as a major limitation. Also, early studies often included small and/or heterogeneous cohorts of patients with respect to clinical stage and grade of the disease, and many results were obtained using standalone PET rather than PET/CT [3,6,7]. More recent clinical data have shown that FDG uptake tends to increase in more aggressive PCa, either recurrent or metastatic [4,5,8,9]. For instance, Beauregard et al. found a detection rate of 69% for FDG-PET/CT, compared to 13% for conventional imaging (CT and bone scan) in 16 patients evaluated for staging or restaging of non-low-risk PCa [9]. Furthermore, overexpression of glucose transporters has been evidenced in high-Gleason score PCa [10,11]. We therefore hypothesized that FDG-PET/CT might be useful in the initial staging and prognostication of high-grade PCa (Gleason ≥8) at biopsy.

## Methods

### Patients

Fifty-four patients newly diagnosed with a Gleason sum ≥8 adenocarcinoma of the prostate at 12-core transrectal ultrasound-guided prostate biopsy were referred for a staging FDG-PET/CT at CHU de Québec, in addition to whole-body bone scan. Patients with a prior history of malignancy within 5 years were excluded. Baseline patient characteristics are summarized in Table 1. The institutional Ethics committee approved this retrospective study.

**Table 1:** **Patient baseline characteristics**

		**Surgical patients (** ***n*** **= 41)**		**Non-surgical patients (** ***n*** **= 13)**		**All patients (** ***n*** **= 54)**		***P-*** **value**
		**No. (%)**	**Median (range)**	**No. (%)**	**Median (range)**	**No. (%)**	**Median (range)**	
**Age, years**			67.5 (48.7-75.5)		65.8 (50.0-82.6)		66.3 (48.6-82.6)	0.77
Biopsy Gleason score*	8	32 (78.0)		4 (30.8)		36 (66.7)		0.005
	9	9 (22.0)		9 (69.2)		18 (33.3)		
**Pretreatment PSA (ng/ml)**			7.0 (1.7-57.0)		15.9 (2.9-263.0)		7.6 (1.67-263.0)	0.006
	<10.0	31 (75.6)		5 (38.5)		36 (66.7)		
	10.0-19.9	9 (22.0)		3 (23.1)		12 (22.2)		
	≥20.0	1 (2.4)		5 (38.5)		6 (11.1)		
**Clinical T stage** ^**†**^	cT1	15 (36.6)		3 (23.1)		18 (33.3)		0.006
	cT2	14 (34.1)		2 (15.4)		16 (29.6)		
	cT3	6 (14.6)		5 (38.5)		11 (20.4)		
	cT4	0 (0.0)		3 (23.1)		3 (5.6)		
	n/a	6 (14.6)		0 (0.0)		6 (11.1)		

PSA = prostate-specific antigen; n/a = not available.

*Based on 2005 International Society of Urological Pathology Modified Gleason System.

^†^Based on American Joint Committee on Cancer, 7th ed.

### FDG-PET/CT

Patients were asked to fast for 6 hours and their glycaemia was checked. FDG-PET/CT was performed approximately 75 minutes after the administration of 300–500 MBq FDG, with oral contrast, from base of skull to upper thighs, on a Biograph 6 PET/CT system (Siemens Healthcare, Erlangen, Germany).

Clinical reporting of FDG-PET/CT was performed by one of three attending nuclear medicine physicians, using a Syngo MI workstation (Siemens Healthcare). The presence or absence of suspicious FDG uptake in the prostate (intraprostatic FDG uptake or IPFU), regional lymph nodes (LNs) and distant sites was extracted from the clinical reports. More than 8 months after the last patient accrual, one nuclear medicine physician (J.M.B.) reviewed the FDG-PET/CT images blinded to the clinical PET/CT report and any clinical data other than the implicit knowledge that all patients had a biopsy-proven high-grade PCa, and scored the IPFU as follows: 0) no focal uptake above background; focal uptake of 1) mild – less than liver, 2) moderate – similar to liver, 3) intense – more than liver, or 4) very intense level – much more than liver. Scores 0 and 1 were considered negative, while scores 2 to 4 were considered positive for significant IPFU. The prostatic maximum standardized uptake value for body weight (SUV_max_) was measured independently, with caution to exclude any urinary activity. SUV_max_ <4.0 was considered negative for IPFU and ≥ 4.0, positive. In one patient, the SUV_max_ was invalid due to an error in uptake time data entry and this patient was excluded from the quantitative analysis.

### Primary treatment

Patients were offered the best of care treatment depending on their co-morbidities, metastatic status and/or preference. When radical prostatectomy (RP) was selected, an extended bilateral pelvic LN dissection (PLND) was performed first. The PLND consisted in the removal of common iliac, internal iliac, external iliac and obturator LNs. Non-surgical patients were treated with androgen deprivation therapy (ADT) alone or in combination with radiation therapy (RT). Patients managed non-operatively and with metastasis on staging FDG-PET/CT were re-imaged after at least 3 months of ADT to evaluate therapeutic response. Lesions with a radiological response/progression consistent with the biochemical evolution were considered true positive.

### Pathological assessment

The following results were extracted from the clinical pathology reports: PCa involvement by sextant, percentage of prostatic tissue involved, pathological stage, pathological Gleason pattern and sum, LNs status and location.

### Statistical analyses

Two validated post-operative prognostic tools that use clinico-pathological data to predict progression-free survival at 5 years were used: the CAPRA-S scores from University of California, San Francisco (UCSF) and the MSKCC nomogram from Memorial Sloan-Kettering Cancer Center [12-15]. Statistical tests were done using SAS v.9.3 software. Univariate analysis was performed using Pearson’s chi-squared and Fisher’s exact tests. Mann–Whitney *U* test was used for two-group comparisons. Spearman correlations were performed for ordinal variables. All analyses were two-sided. A *P*-value ≤0.05 indicated statistical significance.

## Results

### Treatments and follow-up

**Figure 1:**
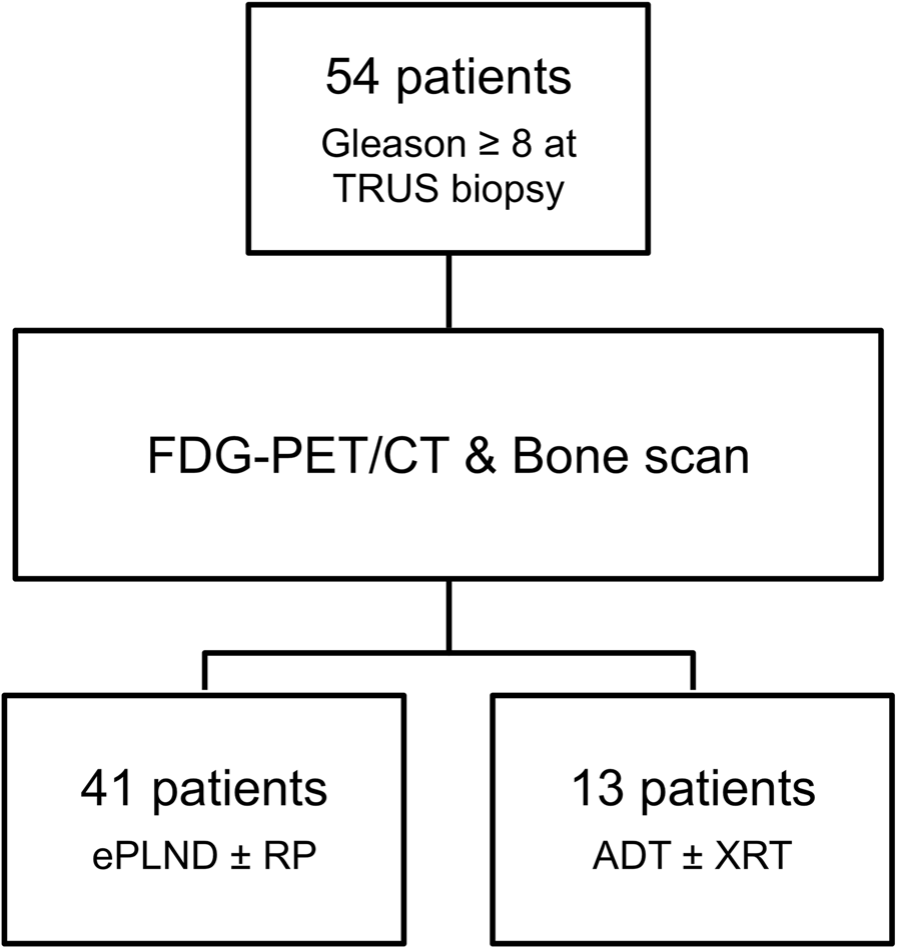
**Study scheme (TRUS = transrectal ultrasound; RP = radical prostatectomy; ePLND = extended pelvic lymph node dissection; ADT = androgen deprivation therapy; XRT = radiation therapy).**

Forty-one patients underwent surgery (Figure 1). Of these, 39 had RP and PLND, while 2 had PLND only due to metastatic LN disease found at the time of surgery. Of the 41 operated patients, 11 (26.8%) were found to harbour LN metastasis at pathology. The remaining 13 patients were treated with ADT, with or without RT. Baseline characteristics of the surgical and the non-surgical groups are compared in Table 1. Pathological data for surgical patients are summarized in Table 2. Among the 39 patients who underwent RP, the Gleason sum was downgraded from ≥8 at biopsy to ≤7 following RP in 26 patients (66.7%). All patients were followed-up for a median of 20 months (range: 15 to 24 months).

**Table 2:** **Pathological characteristics of surgical patients (** ***n*** = 41)*

		**No. (%)**
**Gleason score** ^**†**^	6	3 (7.7)
	7	23 (59.0)
	8	9 (23.1)
	9	4 (10.3)
**Gleason pattern** ^**†**^	3 + 3	2 (5.1)
	3 + 4	8 (20.5)
	3 + 5	1 (2.6)
	4 + 3	16 (41.0)
	4 + 4	8 (20.5)
	4 + 5	3 (7.7)
	5 + 4	1 (2.6)
**Pathological T stage** ^**‡**^	pT2a	1 (2.4)
	pT2b	3 (7.3)
	pT2c	15 (36.6)
	pT3a	9 (22)
	pT3b	10 (24.4)
	pT4a	1 (2.4)
	pT4b	0 (0.0)
	pTx	2 (4.9)
**Margins**	Negative	23 (59.0)
	Positive	16 (41.0)
**Extracapsular extension**	No	21 (53.8)
	Yes	18 (46.2)
**SV invasion**	No	28 (71.8)
	Yes	11 (28.2)
**Perineural invasion**	No	5 (12.8)
	Yes	19 (48.7)
	n/a	15 (38.5)
**Intraprostatic cancer extent** (Mean % (SD))	-	27.3 (22.9)
**Pathological N stage** ^**‡**^	pN0	30 (73.2)
	pN1	11 (26.8)
**Number of LN** (Mean (SD))	-	17.6 (7.3)
**LN density** (Mean (SD))	-	3.6 (8.3)

LN = lymph nodes; SD = standard deviation; SV = seminal vesicles; n/a = not available.

*39 patients underwent radical prostatectomy with extended pelvic lymph node dissection and 2 patients underwent extended pelvic lymph node dissection only.

^†^Based on 2005 International Society of Urological Pathology Modified Gleason System.

^‡^Based on American Joint Committee on Cancer, 7th ed.

### FDG-PET/CT

**Figure 2:**
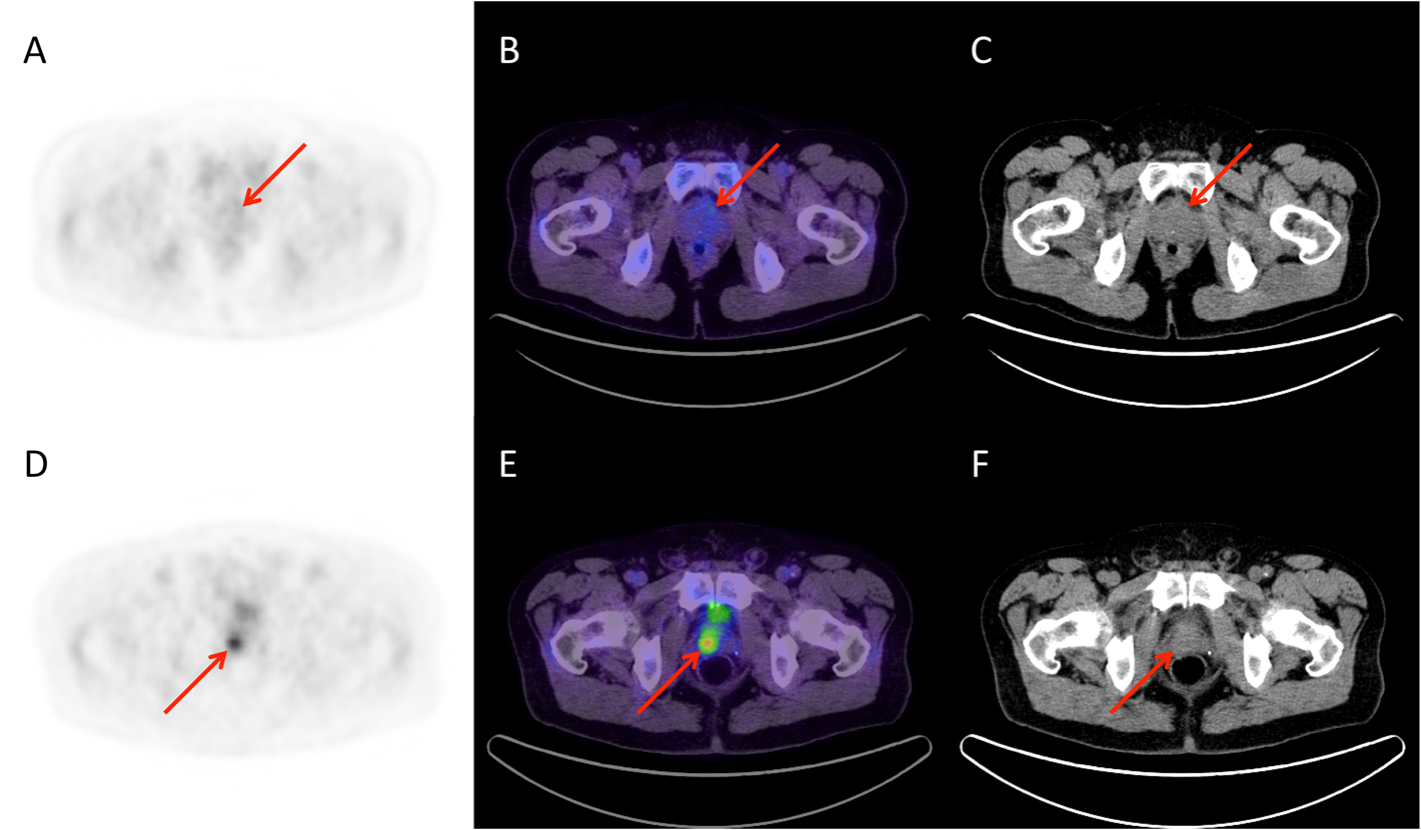
**Examples of corresponding transaxial PET (A, D), fused PET/CT (B, E) and CT (C, F) slices.** A patient **(A-C)** had a Gleason sum 8 (4 + 4) PCa at biopsy and the most prominent focus of prostatic FDG uptake was only faintly noticeable (negative clinical report; Score = 1; SUV_max_ = 2.7). His PCa was downgraded to Gleason sum 6 (3 + 3) after RP. Conversely, another patient **(D-F)** also had a Gleason sum 8 (4 + 4) PCa at biopsy, but FDG-PET/CT showed a highly hypermetabolic prostatic focus (positive clinical report; Score = 3; SUV_max_ = 8.2). His PCa was upgraded to Gleason sum 9 (5 + 4) following RP.

**Figure 3:**
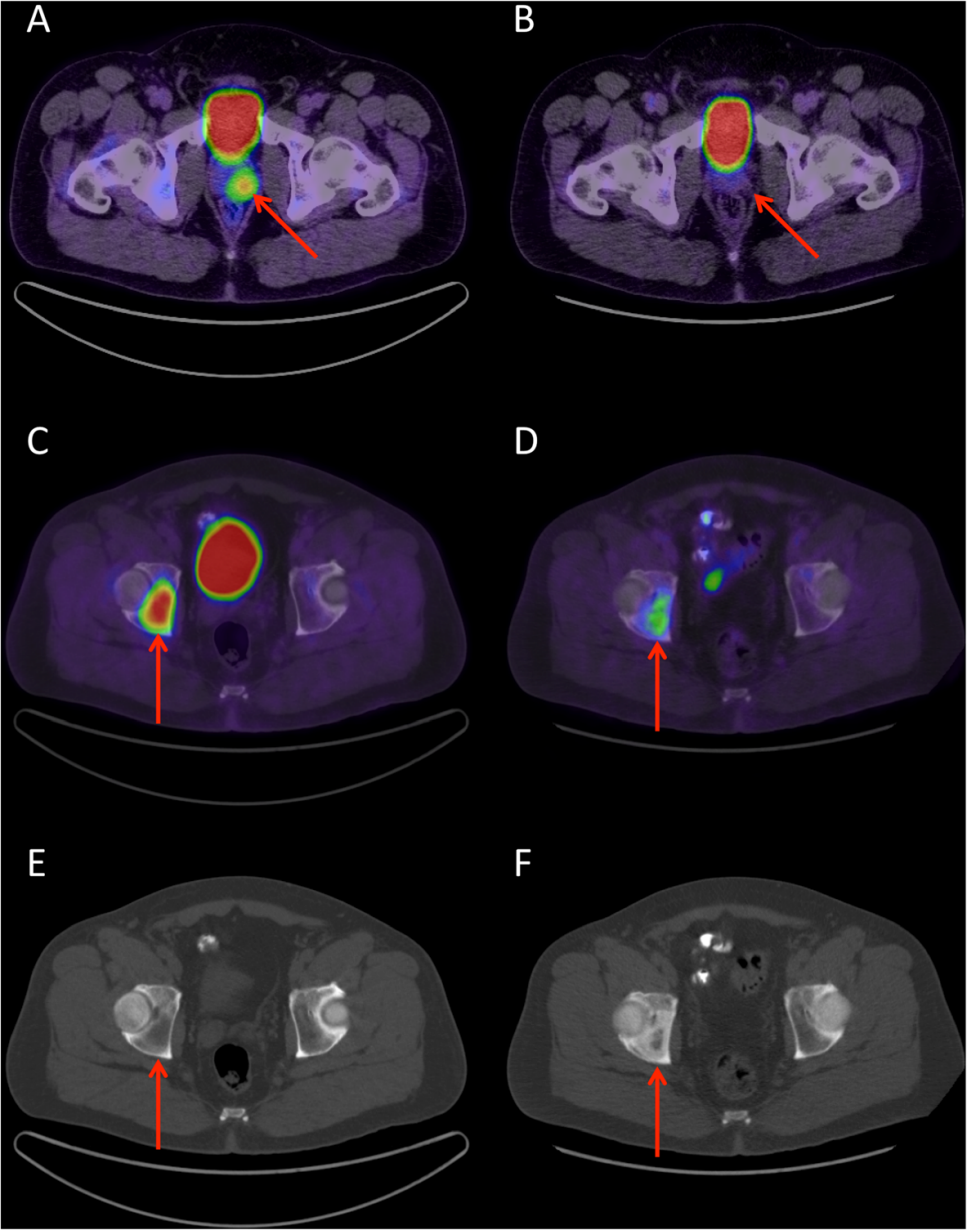
**Fused FDG-PET/CT transaxial slices in a patient with a Gleason sum 9 (4 + 5) PCa at biopsy showing (A) a highly hypermetabolic prostatic focus (positive clinical report; score = 3; SUV**_**max**_ **= 7.1) and (C) one of two bone metastases, which were intensely hypermetabolic and lytic on CT (E).** Three months after ADT initiation, there was a complete metabolic response of the primary PCa lesion **(B)**. The bone lesions underwent at least a partial metabolic response **(D)** and became sclerotic on CT **(F)**. Possibly, the osteoblasts repair activity contributed to the residual FDG uptake. The metabolic response was consistent with the biochemical response, with the PSA decreasing from 125 to 1.5 ng/L.

FDG-PET/CT results from clinical reports, blinded qualitative reading and quantitative analysis are presented on a per-patient basis in Table 3 and examples are depicted in Figure 2. The three patients with bone disease had their bone metastases detected by both FDG-PET/CT and bone scan (Figure 3). Of 7 patients with suspected LN metastasis on FDG-PET/CT, 3 had pathological confirmation at surgery and 4 had metabolic response after 3 months of ADT, consistent with a specificity of 100%. FDG-PET/CT detected LN metastases in 3/11 (27%) patients with pathology-proven LN disease at surgery.

**Table 3:** **FDG-PET/CT results**

	**All patients (** ***n*** **= 54)**	**Surgical patients (** ***n*** **= 41)**	**Non-surgical patients (** ***n*** **= 13)**
	***No. (%)***	***No. (%)***	***No. (%)***
**Clinical report**			
IPFU+	24 (44.4)	15 (36.6)	9 (69.2)
Lymph node metastasis	7 (13.0)	3 (7.3)	4 (30.8)
Bone metastasis	3 (6.0)	0 (0.0)	3 (23.1)
Blinded reading*			
IPFU score 0	6 (11.1)	6 (14.6)	0 (0.0)
IPFU score 1	18 (33.3)	15 (36.6)	3 (23.1)
IPFU score 2	14 (25.9)	12 (29.3)	2 (15.3)
IPFU score 3	10 (18.5)	6 (14.6)	4 (30.8)
IPFU score 4	6 (11.1)	2 (4.9)	4 (30.8)
IPFU+ (score 2 to 4)	30 (55.6)	20 (48.9)	13 (76.9)
**Quantitative analysis**			
IPFU+ (SUV_max_ ≥ 4.0)	24 (44.4)	15 (36.6)	9 (69.2)
	*Median (range)*	*Median (range)*	*Median (range)*
Prostatic SUV_max_	3.7 (1.8 – 34.7)	3.5 (1.8 – 24.9)	5.9 (2.5 – 34.7)

FDG = ^18^F-fluorodeoxyglucose; IPFU = intraprostatic FDG uptake; IPFU + = IPFU-positive; SUV_max_ = maximum standardized uptake value.

*Blinded FDG-PET/CT reading resulted in exactly the same detection rates of lymph node and bone metastasis as the clinical reading.

### IPFU vs. clinico-pathological characteristics

The associations between 10 clinico-pathological features and IPFU status are presented in Table 4. IPFU as described in clinical reports was statistically significantly associated with clinical stage, pathological Gleason sum, pathological Gleason pattern and the percentage of prostatic tissue involved by PCa (Table 4). Based on clinical reporting, negative IPFU conferred patients a 84.6% probability of being downgraded to Gleason sum ≤7 at RP, which contrasts with a downgrading probability of 30.8% when IPFU was reported (Table 5). Moreover, all cases of Gleason sum 9 PCa post-RP did have IPFU, while none of the primary Gleason score 3 did.

**Table 4:** **Clinico-pathological features associated with intraprostatic FDG uptake**

		**Clinical reporting**			**Quantitative analysis**		
		**IPFU-**	**IPFU+**	***P*** **-value**	**SUV**_**max**_ **<4.0**	**SUV**_**max**_ **≥4.0**	***P*** **-value**
		**No. (%)**	**No. (%)**		**No. (%)**	**No. (%)**	
Clinical T stage*	cT1	12 (48.0)	6 (26.1)	0.042	12 (50.0)	6 (27.3)	0.13
	cT2	10 (40.0)	6 (26.1)		9 (37.5)	7 (31.8)	
	cT3	3 (12.0)	8 (34.8)		3 (12.5)	7 (31.8)	
	cT4	0 (0.0)	3 (13.0)		0 (0.0)	2 (9.1)	
**Pretreatment PSA (ng/ml)**	<10.0	23 (76.7)	13 (54.2)	0.21	23 (79.3)	12 (52.2)	0.12
	10.0-19.9	5 (16.7)	7 (29.2)		4 (13.8)	7 (30.4)	
	≥20.0	2 (6.7)	4 (16.7)		2 (6.9)	4 (17.4)	
Pathological T stage*	pT2	14 (53.8)	5 (38.5)	0.28	14 (58.3)	5 (35.7)	0.17
	pT3	12 (46.2)	7 (53.8)		10 (41.7)	8 (57.1)	
	pT4	0 (0.0)	1 (7.7)		0 (0.0)	1 (7.1)	
Pathological N stage*	pN0	21 (80.8)	9 (60.0)	0.15	21 (84.0)	9 (60.0)	0.090
	pN1	5 (19.2)	6 (40.0)		4 (16.0)	6 (40.0)	
**Gleason score** ^**†**^	6	3 (11.5)	0 (0.0)	0.002	3 (12.5)	0 (0.0)	0.029
	7	19 (73.1)	4 (30.8)		17 (70.8)	6 (42.9)	
	8	4 (15.4)	5 (38.5)		4 (16.7)	5 (35.7)	
	9	0 (0.0)	4 (30.8)		0 (0.0)	3 (21.4)	
**Gleason pattern** ^**†**^	3 + 3	2 (7.7)	0 (0.0)	0.009	2 (8.3)	0 (0.0)	0.062
	3 + 4	8 (30.8)	0 (0.0)		7 (29.2)	1 (7.1)	
	3 + 5	1 (3.8)	0 (0.0)		1 (4.2)	0 (0.0)	
	4 + 3	12 (46.2)	4 (30.8)		11 (45.8)	5 (35.7)	
	4 + 4	3 (11.5)	5 (38.5)		3 (12.5)	5 (35.7)	
	4 + 5	0 (0.0)	3 (23.1)		0 (0.0)	2 (14.3)	
	5 + 4	0 (0.0)	1 (7.7)		0 (0.0)	1 (7.1)	
**Intraprostatic cancer extent**	0-9%	8 (30.8)	0 (0.0)	0.027	8 (33.3)	0 (0.0)	0.046
	10-49%	15 (57.7)	7 (58.3)		13 (54.2)	9 (69.2)	
	≥50%	3 (11.5)	5 (41.7)		3 (12.5)	4 (30.8)	
**Margins**	Negative	15 (57.7)	8 (61.5)	0.82	15 (62.5)	8 (57.1)	0.74
	Positive	11 (42.3)	5 (38.5)		9 (37.5)	6 (42.9)	
**Extracapsular extension**	No	15 (57.7)	6 (46.2)	0.50	15 (62.5)	6 (42.9)	0.24
	Yes	11 (42.3)	7 (53.8)		9 (37.5)	8 (57.1)	
**SV invasion**	No	20 (76.9)	8 (61.5)	0.31	19 (79.2)	8 (57.1)	0.15
	Yes	6 (23.1)	5 (38.5)		5 (20.8)	6 (42.9)	

FDG = ^18^F-fluorodeoxyglucose; IPFU = intraprostatic FDG uptake; IPFU- = IPFU-negative; IPFU+ = IPFU-positive; PSA = prostate-specific antigen; SV = seminal vesicles.

*Based on American Joint Committee on Cancer, 7th ed.

^†^Based on 2005 International Society of Urological Pathology Modified Gleason System.

**Table 5:** **Intraprostatic FDG uptake as a predictor of pathological Gleason sum**

	**Pathological Gleason sum**		**Spearman** ***r***	***P*** **-value**
	**≤7**	**≥8**		
	**No. (%)**	**No. (%)**		
**Clinical reporting**				
IPFU-	22 (84.6)	4 (15.4)		0.003
IPFU+	4 (30.8)	9 (69.2)		
**Blinded reading**				
IPFU- (Score 0 or 1)	17 (85.0)	3 (15.0)		0.013
IPFU+ (Score 2 to 4)	9 (47.4)	10 (52.6)		
Score vs. post-RP Gleason pattern			0.58	0.0001
Score vs. post-RP Gleason sum			0.50	0.001
Score vs. pathological T stage			0.32	0.040
**Quantitative analysis**				
IPFU- (SUV_max_ < 4.0)	20 (83.3)	4 (16.7)		0.010
IPFU+ (SUV_max_ ≥ 4.0)	6 (42.9)	8 (57.1)		
SUV_max_ vs. post-RP Gleason pattern			0.46	0.004
SUV_max_ vs. post-RP Gleason sum			0.44	0.006
SUV_max_ vs. pathological T stage			0.35	0.030

FDG = ^18^F-fluorodeoxyglucose; IPFU = intraprostatic FDG uptake; IPFU- = IPFU-negative; IPFU + = IPFU-positive; RP = radical prostatectomy; SUV_max_ = maximum standardized uptake value.

Similarly, quantitative IPFU based on SUV_max_ was statistically significantly associated with pathological Gleason sum and percentage of intraprostatic cancer (Table 4). SUV_max_ was significantly higher in patients with Gleason sum ≥8 than those with Gleason sum ≤7 at final pathology (6.62 ± 6.25 vs. 3.53 ± 1.32, respectively; *P* = 0.020), and SUV_max_ ≥4 was significantly associated with pathological Gleason sum ≥8 (Table 5). Conversely, in 39 patients who underwent RP, low IPFU (SUV_max_ <4.0) conferred patients an 83.3% probability of the Gleason sum being downgraded from ≥8 at biopsy to ≤7 at pathology. This probability was 85.0% when using qualitative IPFU assessment from the blinded reading (Table 5).

**Figure 4:**
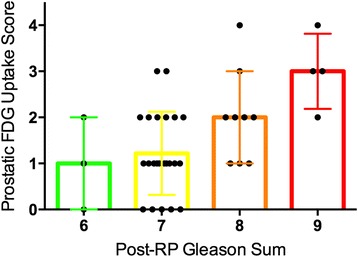
**Prostatic FDG uptake vs. post-RP Gleason sum.**

Statistically significant positive correlations were found between SUV_max_ and visual uptake score on one hand, and post-RP Gleason pattern, Gleason sum and pathological T stage on the other hand (Table 5 and Figure 4).

### IPFU vs. post-radical prostatectomy prognosis

IPFU was compared with the predicted 5-year progression-free survival, as determined by the CAPRA-S and MSKCC nomograms (Table 6) [12-15]. There was a statistically significant difference in predicted 5-year progression-free survival between patients with or without significant IPFU.

**Table 6:** **Intraprostatic FDG uptake as a predictor of the predicted 5-year progression-free survival by CAPRA-S and MSKCC nonograms**

	CAPRA-S*		**MSKCC** ^**†**^	
	**Median % (25th – 75th percentile)**	***P*** **-value**	**Median % (25th – 75th percentile)**	***P*** **-value**
**Clinical reporting**				
IPFU-	70.2 (26.7 – 85.2)	0.008	91.5 (77.0 – 97.0)	0.004
IPFU+	25.9 (0.0 – 42.5)		59.0 (42.0 – 88.0)	
**Blinded reading**				
IPFU- (Score 0 or 1)	70.2 (34.2 – 91.0)	0.017	93.0 (81.0 – 97.5)	0.010
IPFU+ (Score 2 to 4)	26.9 (0.0 – 63.3)		73.0 (45.3 – 89.8)	
**Quantitative analysis**				
IPFU- (SUV_max_ < 4.0)	70.2 (26.7 – 85.2)	0.030	91.5 (72.8 – 97.0)	0.020
IPFU+ (SUV_max_ ≥ 4.0)	25.9 (0.0 – 42.5)		72.0 (45.0 – 89.0)	

FDG = ^18^F-fluorodeoxyglucose; IPFU = intraprostatic FDG uptake; IPFU- = IPFU-negative; IPFU + = IPFU-positive; SUV_max_ = maximum standardized uptake value.

*University of California, San Franciso Cancer of the Prostate Risk Assessment score: Post-Radical Prostatectomy nomogram.

^†^Memorial Sloan-Kettering Cancer Center post-radical prostatectomy nomogram.

## Discussion

Treatment decision after PCa diagnosis is complicated by the variability of disease progression and the diversity of treatments available. To predict PCa behaviour, a number of clinical tools have been developed. One of the simplest is the D’Amico’s risk definition [1]. Most guidelines are based on this classification for treatment recommendations [16,17]. However, the high-risk category includes a wide range of tumour volumes and PSA levels, and also biologically heterogeneous tumours, some being high-grade, highly aggressive tumours (Gleason 9–10), while other being downgraded to Gleason ≤7 at post-RP pathology. Indeed, for high-risk PCa, there is a need for better pre-treatment disease characterization to optimize treatment strategy. Staging is of prime importance in this setting because loco-regional treatment with RP and/or RT is rarely curative when there is LN metastasis. But radiological size-based LN metastasis detection has a poor accuracy [18].

In the last two decades, a number of PET radiopharmaceuticals have been developed for PCa. Of these, ^11^C-choline and ^18^F-fluorocholine (FCH) have been the most studied clinically [19]. Choline radiopharmaceuticals are reputed having a higher uptake in PCa cells than FDG [19], although this is not well established specifically for high-grade PCa. Recent studies in large cohorts have shown promising results for FCH-PET/CT as a staging tool. For example, Beheshti et al. have shown that FCH-PET sensitivity and specificity for PCa LN metastasis detection were 66% and 96%, respectively [20]. Therefore, by comparing these results to ours (27% LN detection sensitivity in surgical patients, 47% overall), it seems that FCH may be superior to FDG for PCa LN staging. More recently, Haseebuddin et al. studied the role of ^11^C-acetate-PET/CT in the primary staging of intermediate and high-risk PCa with negative conventional imaging [21]. They reported a significant difference in 3-year treatment failure probability of 82 and 51%, respectively, when metastasis was found or not, respectively, on pre-operative ^11^C-acetate imaging. One could suggest that LN positivity on FCH- or ^11^C-acetate-PET would carry a greater prognostic value than IPFU, but this is questionable. In fact, reported recurrence-free status at 5 years in patients harbouring pN1 disease (without adjuvant ADT) is approximately 20 to 26% [22,23]. This is similar to the percentage of patients predicted to be disease-free at 5 years by CAPRA-S nomogram when IPFU is present, which was 26.9% in our series. Moreover, 27% (3/11) of pathologically-proven LN metastasis were identified by FDG-PET/CT and the two patients for whom CAPRA-S calculation was possible (one did not undergo RP) had a predicted 5-year recurrence-free survival of 0%. This shows the potentially enhanced discriminative prognostic capability of the widely available FDG radiopharmaceutical compared with choline or acetate tracers.

Moreover, in our series, FCH-PET would theoretically have identified only 7 patients with LN metastasis (66% of 11 pN1) while FDG-PET identified 15 patients with a high local treatment failure probability based on IPFU. Indeed, the tumour biology (or intrinsic aggressiveness) might be as important to consider pre-operatively as LN staging, since some aggressive cancers will metastasize systemically, skipping LN transition. This is consistent with the observation that 2 out of our 3 patients with bone metastasis had IPFU and FDG-positive bone metastasis without any evidence of LN metastasis. In their series of metastatic castration-resistant PCa (CRPC) imaged by FDG-PET, Jadvar et al. observed that 41.4% of their patients had bone-only metastases while 5.7% had both bone and soft tissue metastases [5]. This suggests that radiological and/or surgical LN staging is insufficient for prognostic stratification of high-grade PCa patients. Taken together, these results suggest that FDG-PET/CT, as a single imaging modality, can identify many patients at high risk of local treatment failure, possibly with better discriminative prognostic capability than choline- or acetate-PET. Prospective trials directly comparing FDG with these tracers in patients with high-grade PCa at biopsy are warranted to ascertain this hypothesis.

The prognostic capability of FDG-PET/CT in high-grade PCa is not counterintuitive. The prognostic value of FDG-PET/CT was shown in patients with metastatic PCa by Meirelles et al., who reported that FDG uptake in metastatic lesions of 51 patients (39 CRPC and 12 castration-sensitive) was correlated to prognosis [4]. Jadvar et al. reported that the summed FDG uptake (SUV_max_) of metastatic lesions could predict overall survival in a large cohort of 87 CRPC patients [5]. In our series, higher metabolic activity of the primary PCa correlated strongly with higher pathological Gleason sum and predicted nomogram-derived prognosis. Together, these results suggest that PCa with higher glucose metabolism is associated with a poorer prognosis, both at early and late stages of the disease evolution. Whether increased FDG uptake is maintained throughout the course of the disease or acquired after clonal selection under ADT remains to be elucidated, but early identification of IPFU might prompt a more aggressive systemic management, even in apparently localized PCa.

A unique feature of FDG-PET when compared to choline-PET is its ability to predict post-RP Gleason sum and pattern. Most choline-PET studies did not find any correlation between intraprostatic choline uptake and Gleason sum [24-26]. In our study, negative IPFU indicated an 84.6% probability that a patient would be downgraded to Gleason ≤7 at RP. Moreover, there was a significant correlation between IPFU (SUV_max_ and score) and post-RP Gleason pattern and sum. A potential role for FDG-PET/CT could be the assessment of IPFU to guide adjuvant ADT duration decisions when RT is the primary treatment. The optimal length of ADT in D’Amico’s high-risk patients is still debated, and it may be that IPFU-negative patients could be suitable for shorter ADT duration. FDG-PET/CT could also prospectively identify patients for whom peri-operative chemotherapy or new ADT drugs could be beneficial.

Our study has some limitations. Firstly, the clinical reporting of FDG-PET/CT may have been biased by the variability among attending nuclear medicine physicians in their interpretation of what constitutes significant or suspicious IPFU. However, the systematic blinded reading with qualitative IPFU assessment and the quantitative analysis both corroborate the results derived from clinical reporting. Secondly, most conclusions are based on the surgical subgroup. Because treatment decision was left to the surgeon and the patient (standard of care), many cases of FDG-positive LN or bone metastasis and/or unresectable disease could not be verified pathologically. Since all of them had a metabolic response to ADT after reimaging at 3 months, their metastatic status was probably true positive. Likely, the LN sensitivity of 27% in our surgical group is an underestimation of the overall sensitivity of FDG for detection of LN metastasis, which may be closer to 47% (7/15, when including the 4 non-surgical patients with FDG-positive LN). Finally, IPFU’s prognostic ability was shown indirectly using two prognostic nomograms, the CAPRA-S and the MSKCC. Certainly, the gold standard to demonstrate the prognostic value of FDG-PET/CT will be our cohort’s actual progression-free survival, which will be assessed in the upcoming years. However, we are confident that the estimated prognostic ability of FDG-PET/CT is real since it is based on two of the most validated prognostic tools [12-15]. Moreover, we have shown that IPFU was associated with higher pathological Gleason sum and pattern, and percentage of intraprostatic cancer, all of which have been described as being of prognostic value [15,27,28]. Hence, it is highly expected that actual progression-free survival will be different between low/negative and high/positive IPFU, as the latter might represent the integration of these poor-prognosis pathological features that are only known after surgery.

## Conclusions

Our results suggest that molecular imaging of patients with high-grade PCa at biopsy using FDG-PET/CT could be useful for both staging and prognostic stratification. Intraprostatic FDG uptake assessment with PET/CT may represent the integration of a number of important pathological features, making this crucial prognostic information available before primary therapy. Hence, FDG-PET/CT has the potential to enable improved and personalized care management in this selected PCa patient population, which is most at risk of therapy failure and shortened survival.
